# Genome Sequence of a Gram-Positive Diazotroph, *Paenibacillus durus* Type Strain ATCC 35681

**DOI:** 10.1128/genomeA.00005-16

**Published:** 2016-02-18

**Authors:** Mardani Abdul Halim, Ahmad Yamin Rahman, Kee-Shin Sim, Hok-Chai Yam, Ainihayati Abdul Rahim, Amir Hamzah Ahmad Ghazali, Nazalan Najimudin

**Affiliations:** School of Biological Sciences, Universiti Sains Malaysia, Penang, Malaysia

## Abstract

Here, we report the complete genome sequence of *Paenibacillus durus* type strain ATCC 35681, which can fix atmospheric nitrogen even in the presence of nitrate.

## GENOME ANNOUNCEMENT

*Paenibacillus durus* ATCC 35681^T^ is a nitrogen-fixing bacterium and can be found as free-living or in a symbiotic relationship with plant roots. Here, we report the complete genome sequence of *P. durus* ATCC 35681^T^, a unique strain which can fix atmospheric nitrogen even in the presence of nitrate (1). Members of this species are normally found within the maize, sugarcane, wheat, and sorghum rhizospheres (2). It was previously known as *Bacillus azotofixans, Paenibacillus azotofixans*, and *Paenibacillus durum* before its taxonomic status was revised to its current name of *P. durus* (3, 4).

In a previous study, three *nifH*-homologues were reported in *P. durus* (5). The occurrence of multiple *nifH* genes within a single organism suggested that they serve to modulate nitrogenase activity under certain environmental conditions (6). Such traits make the study of the regulation of the multiple *nifH* genes of *P. durus* under varying environmental conditions and its adaptation to different ecological niches interesting.

The genome was sequenced using the Pacific Biosciences RS (PacBio) and Illumina HiSeq2000 platforms. A 10-Kb single-pass library was performed for PacBio while for the Illumina platform, a 500 bp paired-end library was used. These resulted in 14-fold and 110-fold coverage for the PacBio and Illumina platforms, respectively. PacBio genome data was assembled using the “RS_HGAP_Assembly.2” protocol included in SMRT Portal version 2.3.0. The Illumina reads were used for error correction. CONTIGuator version 2.7, a bacterial genome finishing software tool, was used to re-arrange and join all the contigs into one scaffold (7, 8). PCR primers were subsequently developed to close any remaining gaps between the scaffolds by DNA amplification and sequencing.

The genome sequence was annotated using the NCBI Prokaryotic Genome Annotation Pipeline to predict protein coding genes, structural RNAs, small noncoding RNAs, and tRNAs (9). Additional annotations were performed using the RAST server (SEED and rapid annotation tools) (10) and BASys (11).

The genome of *P. durus* ATCC 35681^T^ consists of a circular chromosome of 5,575,484 bp in length and a G+C content of 51%. No plasmid was detected. Among the 5,233 predicted genes, 4,808 were identified as protein coding genes and 125 were RNAs. From the assigned protein coding genes, 2,904 has putative function while the remaining 1,904 genes were annotated as hypothetical proteins.

Spore formation and nitrogen-fixation genes were detected in the genome of *P. durus* ATCC 35681^T^. The genome harbors six copies of *nifH* homologs and one of these encoded an alternative dinitrogenase reductase (5, 12). This phenomenon of having multiple copies of *nifH* has been reported in various diazotrophs such as *Clostridium pasteurianum*, which likewise has six copies of *nifH*, one of which codes for the alternative vanadium-containing nitrogenase (6, 13, 14). The *nifH1* homolog lies within a nitrogen fixation gene cluster consisting of *nifB1, nifH1, nifD1, nifK, nifE, nifN, nifX, hesA, nifV* resembling the arrangement in *Paenibacillus* sp. WLY78 (15).

### Nucleotide sequence accession number.

The genome sequence of *P. durus* ATCC 35681^T^ has been deposited in GenBank under the accession number CP011114.
